# Testing the feasibility of 30 min of daily outdoor office work for stress management: A pilot study

**DOI:** 10.1177/10519815251356168

**Published:** 2025-07-10

**Authors:** Per Andreasson, Susanna Toivanen, Feben Javan Abraham, Katarina Bälter

**Affiliations:** 1 School of Health, Care and Social Welfare, Division of Psychology, Mälardalen University, Eskilstuna, Sweden; 2 School of Health, Care and Social Welfare, Division of Sociology, Mälardalen University, Västerås, Sweden; 3 School of Health, Care and Social Welfare, Division of Public Health Sciences, Mälardalen University, Västerås, Sweden; 4 Department of Medical Epidemiology and Biostatistics, Karolinska Institutet, Stockholm, Sweden

**Keywords:** health promotion, occupational health, occupational stress, working conditions, pilot projects, environmental psychology

## Abstract

**Background:**

Perceived stress in the workplace is a growing concern, with modern office environments often contributing through noise, crowding, and lack of privacy. To support office workers’ well-being and recovery during the workday, new ways of working—such as spending time outdoors—are being explored.

**Objective:**

This explorative pilot study aims to investigate whether engaging in at least 30 min of outdoor office work each day for five consecutive working days could reduce self-rated stress levels among office workers.

**Methods:**

A within-subjects design was employed. During the first week, eight participants followed their regular office routine. In the second week, the same participants were instructed to spend a minimum of 30 min each day performing office work outdoors.

**Results:**

In the second week, participants worked outdoors for an average of 322 min per week (≈64 min/day), primarily in urban nature settings. Self-rated stress levels were 15% lower than in the first week, a statistically significant reduction by paired-sample t-test, though not by the Wilcoxon test.

**Conclusions:**

Daily outdoor work is feasible among office workers and may reduce self-rated stress levels, warranting larger studies with more diverse participants and environments.

## Introduction

Occupational stress is a significant challenge for both individuals and society, underscoring the need for effective preventive measures. For office workers, the indoor environment itself can be a source of stress, with factors such as noise, crowding, and lack of privacy negatively affecting well-being and performance.^1,2^ Spending time outdoors offers a simple way to introduce variety into sedentary office work, while also increasing physical activity and providing exposure to fresh air and natural daylight.^3^ To address stress levels among office workers and support overall well-being, and to facilitate recovery during the workday, there is a growing need to develop new ways of working—such as spending time outdoors.^4–9^ Outdoor office work includes tasks such as planning, meetings, problem-solving, breaks, and reading, all conducted in an outdoor setting.^7^ Although few studies have specifically examined the effects of outdoor office work on office workers, existing research suggests that participants often report feeling more connected to nature, as well as more energized and calm when working outdoors.^5,7,10^ Moreover, it has been suggested that outdoor office work, even in urban spaces, could promote well-being.^4^

Workplace-related studies have increasingly shown that exposure to natural environments can reduce stress and improve mental well-being among employees. A systematic review by Ríos-Rodríguez et al.^11^ examined nature elements in office settings and found that features such as access to green outdoor areas can effectively reduce stress responses in workers. Similarly, Lottrup, Grahn, and Stigsdotter^12^ demonstrated that employees with access to green outdoor environments at their workplace reported lower levels of perceived stress compared to those without such access. Further supporting this, a systematic review by Gritzka et al.^13^ concluded that nature-based interventions in the workplace generally have positive effects on employee mental health, including the reduction of stress. In a broader perspective, Bratman et al.^3^ highlighted the mental health benefits of nature as a vital ecosystem service, reinforcing its relevance for both public health and organizational settings. Findings from Brossoit^14^ and colleagues indicate that individuals living and working in areas with abundant natural features, such as varied landscapes, water, and temperate climates, tend to spend more time outdoors and find greater enjoyment in outdoor activities. These positive experiences, in turn, are linked to increased workplace engagement and enhanced creativity.^14^ Collectively, these findings support the rationale for outdoor office work as a promising approach to integrating restorative nature exposure into daily work routines to alleviate stress and promote employee well-being.

According to the demand-control model,^15^ a widely used framework for understanding job stress, high psychological job demands combined with low job control result in high job strain. The model was later expanded to include social support at work, which can buffer the adverse effects of job strain.^16,17^ This model has been used to predict a range of adverse health outcomes, including psychological well-being,^18,19^ depression,^20^ and cardiovascular disease.^21,22^ Moreover, perceived daily stress levels have in previous studies been reliably measured using a single-item question, validated as an indicator of general stress symptoms.^23^ Prolonged or repeated exposure to stress without sufficient recovery has been found to contribute to allostatic load and stress-related physiological dysregulation.^24,25^

Numerous studies have found that nature experiences reduce stress levels.^26–33^ A systematic review and meta-analysis of 143 studies by Twohig-Bennett and Jones^29^ reported decreased salivary cortisol, heart rate, and diastolic blood pressure after exposure to outdoor environments. Ulrich et al.^26^ demonstrated that recovery from stress was more effective in natural environments compared to urban settings, resulting in a more positive emotional state and lower arousal levels, possibly indicating increased parasympathetic activation, in line with psychoevolutionary theory.^26,34^ The parasympathetic nervous system is activated during rest and involves, among other things, lowering the heart rate and reducing blood pressure.^35^ In this sense, exposure to nature can be regarded as an antidote to stress.

Spending 120 min a week in nature has been associated with a greater likelihood of reporting high well-being and good health, according to White et al.^36^ It did not matter if the time in nature was spread across several shorter periods or one long period. Additionally, Hunter et al.^30^ discovered that the stress-reducing effect of nature experiences, measured by a reduction in salivary cortisol, was most pronounced per time unit in the interval between 20–30 min.

An exercise-based intervention in the workplace to reduce job stress, conducted either outdoors or in an indoor exercise setting, revealed that the outdoor group reported higher perceived potential for restoration and positive affect, along with lower cortisol levels and marginally lower diastolic blood pressure, as found by Calogiuri et al.^37^ This suggests that being outdoors has a stress-reducing effect beyond that of exercise alone.

A comparison of short walks of 20–30 min in urban green settings versus urban grey settings found increased positive mood and higher heart rate variability (HRV) in the green settings, as reported by Neale et al.^38^ A systematic review demonstrated that heightened stress is associated with lowered HRV, specifically linked to decreased parasympathetic activation, as shown by Järvelin-Pasanen et al.^39^ Therefore, the higher HRV observed in urban green settings indicates lower levels of stress.

The aim of the present pilot study was to explore whether engaging in at least 30 min of outdoor office work daily over five consecutive working days could reduce self-rated stress levels in office workers. Outdoor office work could take place in natural environments as well as in urban environments.

## Methods

### Design

A within-subjects experimental design was employed, where participants acted as their own controls. During the first week, participants followed their usual office work routine indoors, and in the second week, they engaged in at least 30 min of outdoor office work every working day.

### Settings

This pilot study was a sub-project of the larger research initiative, “Concepts of the Sustainable Office of the Future” (SOFCO). The SOFCO project aims to promote a healthy working life and lifestyle among office workers, develop concepts for the office of the future, and contribute to sustainable development.^40^ The present sub-project focused on new ways of working in terms of outdoor office work in relation to stress, recovery, and health. The study was conducted in Stockholm, Sweden, between 10 October and 21 October 2022. There was no reward for participating in the study except for individual feedback on the heart rate variability measurement.

### Study participants

Office workers were recruited from the external partner companies collaborating with the SOFCO project, representing various sectors of the office industry, including companies involved in property development, office interior design, and office industry consulting. Information about the study was disseminated via email to contact persons at eight partner companies, which then forwarded the information to their employees. A total of nine participants from three of these companies initially signed up for the study. However, one participant was later excluded because the participant spent more time outdoors in the baseline week than in week two. The final sample consisted of eight participants, including six women and two men aged 29–65 years.

The study's inclusion criteria required participants to be currently active indoor office workers, aged between 25 and 65 years. Exclusion criteria included individuals who were already working outdoors prior to the study, those with non-communicable diseases (NCDs) such as diabetes or heart conditions, and those taking medications, such as beta-blockers, antidepressants, benzodiazepines, or anti-inflammatory drugs, within the two weeks leading up to the study.

### Data collection

#### Questionnaires

Participants completed three web-based questionnaires: a baseline questionnaire on the first day, a follow-up questionnaire on the final day, and a short daily questionnaire each working day. Links to the questionnaires were distributed via email through the Survey Generator system, allowing participants to complete them using either a computer or a smartphone.

The baseline and the follow-up questionnaires encompassed inquiries about working conditions, overall health status, sleep quality, stress levels, and physical activity, requiring approximately 20–25 min to complete. The questions concerning the job demand-control-support model were sourced from the Swedish Longitudinal Survey on Health (SLOSH).^41^ Three indexes were computed within the Job Demand-Control-Support model: Psychological demands at work, Decision latitude at work, and Social support at work. The average score for each index was calculated, the theoretical range was 1 to 4.

In a large sample (n = 5277), the internal consistency (Cronbach's alpha) was found to be .73 for Psychological Demands, .74 for Decision Latitude, and .83 for Social Support using the Swedish Demand-Control-Support Questionnaire in a Norwegian translation.^42^ Furthermore, the factor structure supported the three dimensions, and the intercorrelations between the subscales were low. In addition, the English and German versions have shown Cronbach's alpha ≥ .72 for Psychological Demands, Decision Latitude, and Social Support, and the hypothesized factorial structure with three factors was supported by the results of exploratory factor analysis.^43^

The Psychological demands at work index was calculated from five questions: “Do you have to work very fast?”, “Do you have to work very intensively?”, “Does your work demand too much effort?”, “Do you have enough time to complete your job?” and “Does your work often involve conflicting demands?” The response categories and scores for Demands at work, with the values in parentheses, were: “Often” (4), “Sometimes” (3), “Seldom” (2), or “Never/almost never” (1), or “do not want to state” (non-response). The question “Do you have enough time to complete your job?” was an exception and was scored as: “Often” (1), “Sometimes” (2), “Seldom” (3), or “Never/almost never” (4), or “do not want to state” (non-response). For all questions, higher values indicate higher degree of demands.

The Decision latitude at work index was calculated from six questions: “Do you have the possibility of learning new things through your job?”, “Does your work demand high level of skill or expertise?”, “Does your work require creativity?”, “Do you have to do the same thing over and over again?”, “Do you have a choice in deciding how you do your work?” and “Do you have a choice in deciding what you do at work?” The response categories and scores for Decision latitude at work, with the values in parentheses, were: “Often” (4), “Sometimes” (3), “Seldom” (2), “Never/almost never” (1), or “do not want to state” (non-response). The question “Do you have to do the same thing over and over again?” was an exception and was scored as: “Often” (1), “Sometimes” (2), “Seldom” (3), or “Never/almost never” (4), or “do not want to state” (non-response). For all questions, higher values indicate higher degree of decision latitude.

The Social support at work index was calculated from six questions: “There is a calm and pleasant atmosphere where I work”, “There is a good spirit of unity”, “My colleagues are there for me”, “People understand that I can have a bad day”, “I get on well with my superiors” and “I get on well with my colleagues”. The response categories and scores for Social support at work, with the values in parentheses, were: “Strongly agree” (4), “Mildly agree” (3), “Mildly disagree” (2), “Strongly disagree” (1) and “do not want to state” (non-response). For all questions, higher values indicate higher degree of social support.

The short daily questionnaire consisted of five questions. The first question was if the participants had been working outdoors that day, with the response alternatives “yes”, “no” or “do not want to state”. The second question was “For how long did you work outdoors today?” The response alternatives were in a dropdown list with options from “1 min”, “5 min”, “10 min” and so on up to “8 h”. The third question was an open-ended question: “What kind of office work did you conduct outdoors today?” The fourth question was an open-ended question: “Describe the environment in which you worked outdoors today”. The fifth and final question was “How stressed have you felt during your workday?” with response options 1 “not at all stressed” to 10 “very stressed” in a dropdown list. The short daily questionnaire was sent out at 3 pm each working day and only took 1–2 min to fill out.

### Procedure

During the first week (days 1–5), participants followed their usual work routine, while in the second week (days 8–12), they engaged in at least 30 min of outdoor office work every working day. No assessments were performed during the weekend (days 6 and 7).

On day 1, participants attended a meeting where they received a pedagogical module of one hour about outdoor office work, how to work outdoors effectively, and the benefits of doing so. Additionally, they completed a baseline questionnaire and signed an informed consent form to participate in the study. Later that day, they filled out the first daily questionnaire.

On day 5, participants were interviewed about their experiences with the study thus far. On day 8, an online meeting reminded participants that the next phase of the project had begun, and they were instructed to work outdoors for at least 30 min every working day of the week. On day 12, participants completed the follow-up assessment questionnaire and were interviewed about their overall experiences of outdoor office work and participating in the study.

To standardize the procedure, participants received the same information about the study, and all participants received the surveys at the same time of the day. However, there was some variation in when participants completed the survey, as not all of them read their email and filled it out immediately upon receiving it.

**Figure 1.**
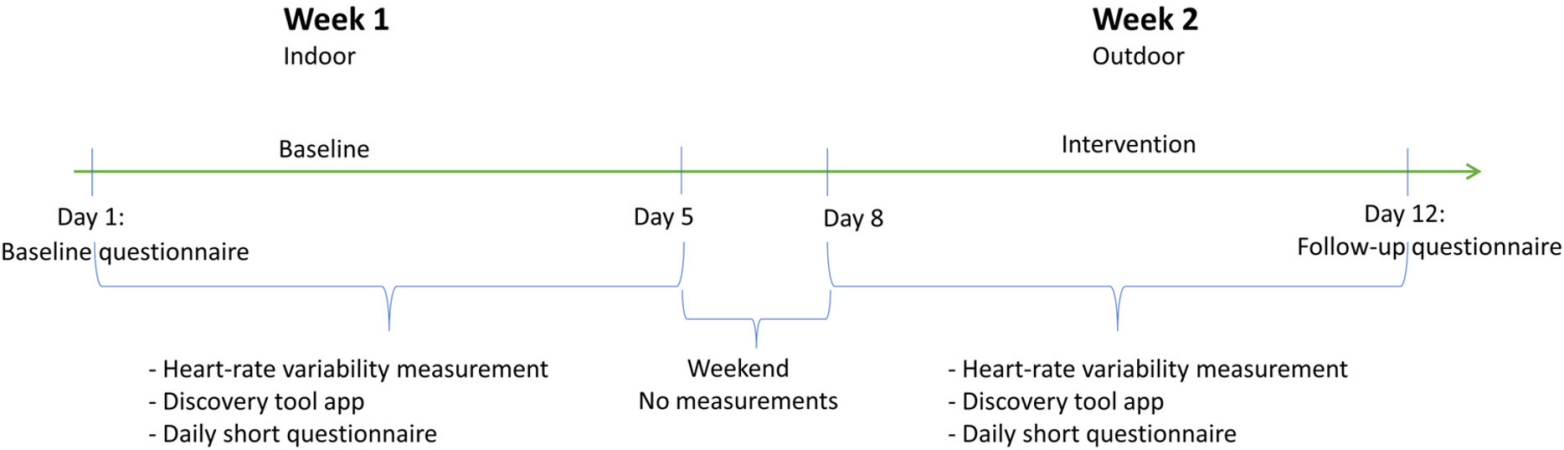
Timeline for data collection, a pilot study in Sweden, 2022.

In addition, participants were instructed to take photos of their working environment and comment on them in an app (Discovery Tool) from day 1 to day 5 and from day 8 to day 12. Furthermore, heart rate variability was measured during the same periods. Process interviews with participants were conducted at the end of both the first and second weeks to assess the feasibility of the pilot study from the participants’ perspective. However, the results from these measures fall beyond the scope of this paper and will be reported elsewhere. For an overview of the procedure, see Figure 1.

### Statistical methods

The mean values of daily stress on a scale from 1 to 10 were calculated for days 1 to 5 (week 1) and days 8 to 12 (week 2). The difference in mean values between week 1 (baseline) and week 2 (intervention) was tested using paired-sample t-test and Wilcoxon Signed Rank Test. Paired-sample t-tests and Wilcoxon Signed Rank tests were also used to assess the differences between the means of Psychological demands, Decision latitude, and Social support at work on a scale from 1 to 4 from baseline (day 1) to the follow-up assessment (day 12). Finally, descriptive statistics of mean values for time spent outdoors (minutes) for week 1 and week 2, respectively, were calculated. Statistical analyses were performed using SPSS version 28, with a significance level set at .05 for all tests.

## Results

Baseline characteristics of the participants are presented in Table 1. The study included eight participants, predominantly women (75%), with only two men (25%). Most participants (50%) were aged between 50 and 59 years, with one participant in each of the other age categories (20–29, 30–39, 40–49, and 60–69 years). Regarding educational background, most participants (88%) held a university degree, while one participant (12%) had completed higher vocational education. No participants reported only elementary or upper secondary education, nor did any report postgraduate education. Self-rated health was evenly distributed: four participants (50%) described their health as “very good” and four (50%) as “fairly good”; no participants reported poorer health statuses. Most participants worked full-time (75%), while two (25%) worked part-time. Reported weekly working hours varied, with most participants (62%) working 40–49 h per week, two (25%) working 30–39 h, and one (13%) working 50–59 h.

**Table 1.** Baseline characteristics of study participants, n = 8.

		N	%
Sex	Men	2	25
	Women	6	75
Age group	20–29	1	12
	30–39	1	12
	40–49	1	12
	50–59	4	50
	60–69	1	12
Education	Elementary school	0	88
	Upper secondary school	0	0
	Higher vocational education	1	12
	University education	7	88
	Postgraduate education	0	0
Health	Very good	4	50
	Fairly good	4	50
	Neither good nor bad	0	0
	Fairly poor	0	0
	Very poor	0	0
Work	Full-time	6	75
	Part-time	2	25
Hours worked per week	30–39	2	25
	40–49	5	62
	50–59	1	12

**Figure 2.**
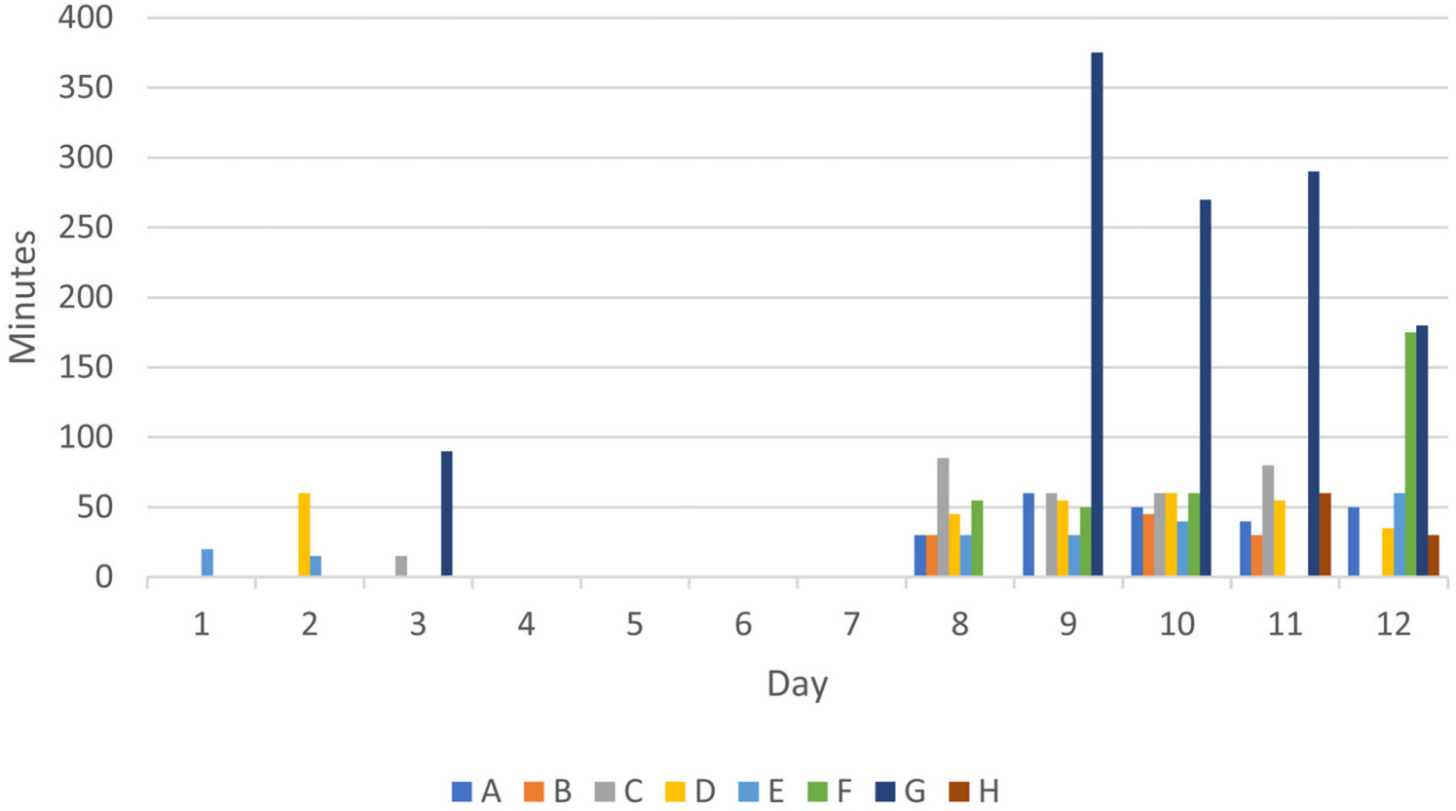
Number of minutes each person conducted outdoor office work each working day during week 1 and week 2. The letters A to H represent individual participants, n = 8.

Figure 2 displays a bar chart illustrating the time spent on outdoor office work, in minutes per day, by eight participants (A–H) over two consecutive workweeks. During Week 1 (Days 1–5), outdoor work was sporadic and limited, with only a few participants recording short durations. In contrast, during Week 2 (Days 8–12), when the intervention was implemented, all participants engaged in outdoor office work more consistently, with most reaching or exceeding the target of 30 min per day. There was notable variability in individual engagement, with some participants reporting substantially longer durations of outdoor office work than others on certain days. These findings indicate increased adherence to outdoor office work during the intervention week, supporting the feasibility of the pilot protocol.

Table 2 presents the average number of minutes participants spent on outdoor office work during Week 1 (Days 1–5) and Week 2 (Days 8–12). All eight participants (A–H) increased their time spent outdoors during the intervention week (Week 2) compared to the baseline week (Week 1). The total average time increased from 25 min/week in Week 1 to 322 min/week in Week 2, representing a mean increase of 297 min/week. This corresponds to an increase from 5 min/day during week 1 to 64 min/day during week 2, almost twice as much as they were instructed to do. On an Individual level, one participant increased the time outdoor from 90 to 1025 min. These findings indicate a clear change in behavior following the implementation of the outdoor office work intervention and that outdoor office work was appreciated.

**Table 2.** Total time spent doing outdoor office work during days 1–5 (week 1) and days 8–12 (week 2) for each participant; the letters A to H represent individual participants, n = 8.

Participant	Week 1 (minutes/week)	Week 2 (minutes/week)	Difference (Week 2–Week 1) (minutes/week)
A	0	230	230
B	0	105	105
C	15	285	270
D	60	250	190
E	35	160	125
F	0	340	340
G	90	1115	1025
H	0	90	90
Mean	25	322	297

Table 3 displays the number of occasions on which participants conducted outdoor office work in various types of environments during Week 1 and Week 2. “Nature” environments could include, for example, forests; “Urban nature” may encompass villa gardens or park settings; and “Urban” refers to typical urban areas such as streets and built-up environments. During Week 2, outdoor office work was most frequently performed in urban nature environments, indicating a preference for semi-natural settings among participants during the intervention period.

**Table 3.** Number of times outdoor office work was performed in different types of environments, n = 8.

Week	Nature	Urban nature	Urban	Unknown
Week 1	0	2	1	3
Week 2	4	20	3	6

**Figure 3.**
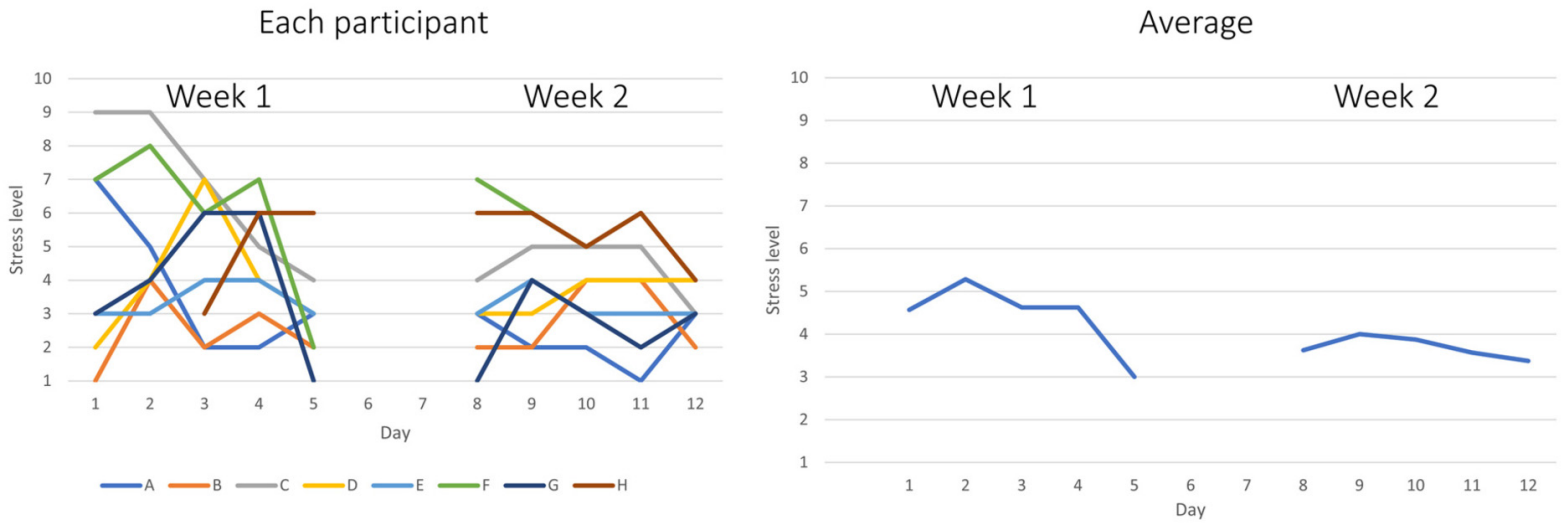
On the left side: self-rated daily stress level for each participant rated from 1–10, where 1 indicates not at all stressed and 10 indicates very stressed. The letters A to H represent individual participants. On the right side: average daily stress levels for week 1 and week 2 for all participants, n = 8.

Figure 3 presents self-rated daily stress levels among participants across two consecutive workweeks. The left panel shows individual stress trajectories for each participant (A–H), rated on a scale from 1 (not at all stressed) to 10 (very stressed). Considerable day-to-day variation is observed in Week 1 (Days 1–5), with some participants reporting relatively high stress levels. In contrast, Week 2 (Days 8–12), the intervention period, displays more stable and generally lower stress ratings across participants. The right panel illustrates the mean daily stress level for all participants (n = 8), indicating a reduction of stress from Week 1 to Week 2. The mean stress level was moderately high during Week 1, with a noticeable decline by Day 5. During Week 2, the mean stress level remained consistently lower, suggesting a potential reduction in perceived stress during the period of daily outdoor office work.

A paired-samples t-test revealed a statistically significant decrease in self-rated stress between the average of the five days in Week 1 (M = 4.42, SD = 1.43) and Week 2 (M = 3.74, SD = 1.31), t(7) = 1.91, p = .049 (one-sided), corresponding to a medium effect size (d = 0.67). This indicates that self-rated stress was 15% lower during Week 2 compared to Week 1. Given the small sample size, a non-parametric Wilcoxon Signed Rank test was also conducted but did not show statistically significant difference between Week 1 (Mdn = 4.00, IQR = 1.55) and Week 2 (Mdn = 3.40, IQR = 1.90), W = 27.50, p = .104 (one-sided).

Tables 4 and 5 present the results of paired-sample t-tests and Wilcoxon Signed Rank tests assessing changes in work-related stress indicators—Psychological Demands, Decision Latitude, and Social Support at work—between Week 1 and Week 2. No statistically significant differences were found for any of the indices.

**Table 4.** Changes in work related stress between week 1 and 2 based on the indices of psychological demands, decision latitude and, social support at work, n = 8, using T-tests.

	Week 1		Week 2		Difference (Week 1–week 2)	*t*_7_	*p* (two-sided)
	Mean	***SD***	Mean	***SD***			
Psychological demands	2.32	0.34	2.55	0.48	−0.23	2.35	.051
Decision latitude	3.35	0.33	3.21	0.19	0.14	2.20	.064
Social support	3.73	0.29	3.69	0.40	0.04	0.61	.563

**Table 5.** Changes in work related stress between week 1 and 2 based on the indices of psychological demands, decision latitude and, social support at work, n = 8, using Wilcoxon signed rank tests.

	Week 1		Week 2		Difference (Week 1–week 2)	*W*	*p* (two-sided)
	Median	*IQR*	Median	*IQR*			
Psychological demands	2.30	0.45	2.50	0.45	−0.20	2.00	.090
Decision latitude	3.33	0.29	3.25	0.33	0.08	19.50	.073
Social support	3.83	0.54	3.92	0.67	−0.09	2.00	.56

## Discussion

The main finding of this pilot study is that self-rated work stress levels were lower during the week in which participants engaged in daily outdoor office work compared to the week in which they followed their usual indoor routine. This was supported by the results of the parametric t-test, although the nonparametric Wilcoxon Signed Rank test did not reach statistical significance. The discrepancy is likely due to the small sample size and the lower statistical power of the nonparametric test.

Participants were instructed to work outdoors for a minimum of 150 min during Week 2 (i.e., 30 min per workday). On average, participants greatly exceeded this requirement, spending 322 min outdoors during Week 2 (corresponding to 64 min per day), compared to only 25 min during Week 1 (or 5 min/day). This substantial increase shows that participants found outdoor office work appealing. This aligns with findings by White et al.,^36^ who reported that spending at least 120 min per week in nature is associated with improved well-being and health, with diminishing returns observed beyond 200–300 min. Although, individual engagement in outdoor office work varied considerably in Week 2, ranging from 90 to 1115 min, indicating differing preferences or possibilities among participants.

Participants were free to choose the setting for their outdoor office work, whether near their regular office, at home, or in transit. Most of the outdoor work occurred in what was categorized as either “nature” or “urban nature” environments, such as villa gardens or parks. Prior studies, including that of Neale et al.,^38^ have highlighted the restorative potential of green spaces, particularly in urban areas. Therefore, the reduction in perceived stress observed in Week 2 may have been influenced not only by being outdoors but also by the presence of natural elements in the work environment.

Regarding psychosocial work environment indices—psychological demands, decision latitude, and social support—no statistically significant changes were observed between the two weeks. According to the job demand-control-support model,^15,21^ a combination of high demands and low control is associated with increased stress, known as job strain. While outdoor office work could theoretically enhance perceived control, the structured requirement to be outdoors may have paradoxically been perceived as an external demand, potentially offsetting this benefit. Additionally, it is plausible that the daily self-assessments of stress captured more immediate responses to environmental changes, whereas the broader constructs assessed by the demand-control-support questionnaire may require a longer study period to show measurable effects.

This study involved eight participants (six women and two men), which is too small a sample to explore gender differences in response to outdoor office work. The participants were generally healthy, with all rating their health as either “very good” or “fairly good.” Furthermore, the sample was highly educated, with seven participants holding a university degree. These factors limit generalizability, and future studies should include more diverse samples to examine potential differences in outcomes by gender, education level, and baseline health. Age-related differences may also be important to explore in larger samples.

The psychoevolutionary theory^26,34^ offers a plausible explanation for the observed stress reduction, suggesting that exposure to nature induces rapid, unconscious positive emotional and physiological responses, including parasympathetic activation. Given that most outdoor office work occurred in urban nature environments, future studies should examine whether different types of outdoor settings (e.g., forests vs. urban streetscapes) have differential impacts on stress.

A major limitation of this pilot study is the small sample size, which restricts statistical power and limits the generalizability of the findings. While the paired-sample t-test indicated a statistically significant reduction in self-rated stress during the intervention week—supporting the feasibility of outdoor office work as a health-promoting intervention—the corresponding nonparametric Wilcoxon test did not reach significance. This discrepancy suggests that the results may be sensitive to the choice of statistical method, likely due to the limited sample size. As such, these findings should be regarded as exploratory and warrant replication in larger and more diverse samples. Additionally, the use of a single-item self-rated measure of stress may introduce the risk of participant bias. Nonetheless, such measures have demonstrated validity in capturing general stress levels in population-based research.^23^ Another limitation concerns the seasonality of data collection, which occurred during autumn—a time when daylight hours and temperatures in Sweden are still relatively favorable. The effects of outdoor office work may differ under colder and darker conditions, which should be explored in future seasonal or longitudinal studies.

## Conclusion

Poor working conditions in modern offices are a well-documented concern, known to negatively impact employees’ well-being and stress levels.^1^ As a result, alternative ways of working—such as incorporating outdoor environments—need to be explored and evaluated. This pilot study represents an early step in that direction. To our knowledge, it is the first study to examine whether performing office work outdoors for part of the workday may reduce stress and we investigated whether spending at least 30 min per day working outdoors over five consecutive workdays would lower self-rated stress levels. The findings suggest that incorporating regular periods of outdoor office work into the workday may reduce perceived stress, with potential benefits for individuals, organizations, and society. Although the sample size was small, the results encourage future studies with more participants, diverse settings and environments over a longer period of time. To facilitate implementation in every-day working life, employers and managers are encouraged to support and enable employees to include outdoor office work in their daily routines.
